# Efficacy and safety of Xihuang pill for gastric cancer: A protocol for systematic review and meta-analysis

**DOI:** 10.1097/MD.0000000000025726

**Published:** 2021-05-14

**Authors:** Junwei Wang, Daorui Hou, Yahui Peng, Jian Xiong, Lu Xiong, Xin Tan

**Affiliations:** a Department of Traditional Chinese Medicine Oncology, Guang’anmen Hospital South District, Beijing; b Department of Traditional Chinese Medicine Oncology, The First People's Hospital of Xiangtan City, Xiangtan, Hunan Province; c Beijing Shahe Hospital, Beijing; d Department of Oncology, Guang’anmen Hospital, Beijing, China.

**Keywords:** gastric cancer, protocol, systematic review and meta-analysis, Xihuang pill

## Abstract

**Background:**

Xihuang pill has been widely applied as a promising adjunctive drug for gastric cance. However, the exact effects and safety of Xihuang pill have yet to be systematically investigated. We aimed to summarize the effificacy and safety of Xihuang pill for the treatment of advanced GC through the meta-analysis, in order to provide scientific reference for the design of future clinical trials.

**Methods:**

The protocol followed Preferred Reporting Items for Systematic Reviews and Meta-Analyses Protocols. Relevant randomized controlled trials were searched from PubMed, the Cochrane Library, Embase, the China National Knowledge Infrastructure, Wanfang Database, Chinese Science and echnology Periodical Database, and Chinese Biomedical Literature Database. Papers in English or Chinese published from their inception to October 2020 will be included without any restrictions. Cochrane Risk of Bias tool will be used to assess the risk of bias of included studies. The RevMan 5.4 and Stata 16.0 software will be applied for statistical analyses. Statistical heterogeneity will be computed by *I*^2^ tests. Sensitivity analysis will be conducted to evaluate the stability of the results. The publication bias will be evaluated by funnel plots and Egger test. The quality of evidence will be assessed by the Grading of Recommendations Assessment, Development and Evaluate system.

**Results:**

The results of our research will be published in a peer-reviewed journal.

**Conclusion:**

The conclusion of this study will provide helpful evidence of the effect and safety of Xihuang pill for the treatment of GC in clinical practice.

**OSF registration number:**

10.17605/OSF.IO/VFJAK.

## 1 Introduction

Gastric cancer (GC) is the fifth most frequently diagnosed cancer and the third leading cause of cancer death around the world.^[1,2]^ According to an epidemiological survey in 2018, there were 1,033,701 new cases of GC and 782,685 deaths related to GC worldwide.^[3]^

Despite recent advances in therapeutic methods including surgery combined with chemotherapy and radiotherapy, the prognosis for advanced GC patients remains very poor.^[3]^ With high incidence and mortality rate, GC causes a serious health burden globally, especially in several Western Asian and Eastern Asia countries (e.g., in Mongolia, Japan and the Republic of Korea).^[4]^ Thus, implementation of a multimodality treatment approach is of great significance to further improve survival.^[5]^

Traditional Chinese medicine is one of the popular alternative treatments for cancer.^[6]^ It is accepted in China to enhance the antitumor effects of conventional therapies, reduce the toxicity of chemotherapy and radiotherapy, alleviate tumor-induced clinical symptoms and cancer pain, and prolong the survival time of postoperational and advanced-stage cancer patients.^[7]^

Xihuang pill, a famous traditional Chinese medicine formulation, has been used to treat tumor-related diseases in China for more than 100 years.^[8,9]^ Xihuang pill, mainly composed of Ru Xiang (*Olibanun*), Mo Yao (*Myrrha*), She Xiang (*Moschus*), and Niu Huang (*Bovis Calculus*), was approved by the National Medical Products Administration (NMPA) for tumor treatment with approval number Z11020073. Some of the compounds found in these ingredients exert multiple antitumor effects and may synergize with the other ngredients. Xihuang pill can also inhibit the growth of tumor cells and cancer stem cells, prevent tumor invasion and angiogenesis, and regulate the tumor microenvironment. It has exhibited antitumor effects on various cancers such as lung cancer,^[8]^ breast cancer,^[10]^ colorectal cancer,^[11]^ non-Hodgkin lymphoma,^[12]^ and liver cancer.^[13]^

With the publication of numbers of trials and clinical studies on Xihuang pill for GC, there is an urgent need for a systematic review to assess the effectiveness and safety of Xihaung pill in treating gastric cancer. Hereby, we will systematically review current available randomized controlled trials (RCTs) to objective comment the efficacy and safety of Xihuang pill in patients with GC. It may provide more relevant information for clinician in clinical practice.

## 2 Methods and analysis

This protocol of systematic review and meta-analysis has been drafted under the guidance of the referred reporting items for systematic reviews and meta-analyses protocols. Moreover, it has been registered on open science framework (OSF) on August 25, 2020. (Registration number: DOI 10.17605/OSF.IO/VFJAK).

### 2.1 Inclusion criteria

#### 2.1.1 Type of study

We will collect all available RCTs on Xihuang pill for the treatment of gastric cancer without language restriction. Articles without sufficient available data, observational studies, Cross-sectional studies, cross-over studies, quasi-RCT, conference abstracts, animal experiments, case reports, literature reviews, and letters will be excluded.

#### 2.1.2 Types of participants

Patients with gastric cancer be included RCTs who were definitely diagnosed, regardless of nationality, race, age, gender, or course of disease.

#### 2.1.3 Types of interventions

Observation group: Xihuang pill is used alone or in combination with other treatment methods. There will be no restriction about the doses and methods of use of intervention. Control group: Other treatments (including any other non Chinese medicine treatment) or combined with fake Chinese medicine. When Xihuang pill used as combinations with other treatments, the control group should also receive the same combination treatments.

#### 2.1.4 Types of outcomes

Primary outcomes: The recurrence-free survival (RFS) of patients will be analyzed as the primary outcome.

Secondary outcomes: The secondary outcomes include overall survival (OS), progression-free survival (PFS), adverse reactions (including nausea/vomiting, hypo-leukemia, neutropenia, fever, liver dysfunction, renal dysfunction, etc), and toxicity grade coded by common toxicity criteria for adverse events.

### 2.2 Search strategy

Two researchers will independently search 3 English and 4 Chinese databases, including PubMed, the Cochrane Library, Embase, the China National Knowledge Infrastructure, Wanfang Database, Chinese Science, and Technology Periodical Database, and Chinese Biomedical Literature Database. All the above databases will be searched from the available date of inception until the latest issue (October 2020). No language or publication restriction will be used. Google scholar, Bing scholar, and Baidu scholar will also be retrieved to find out other related literature. In addition, we will search grey literatures to avoid missing any potential studies, such as dissertations, ongoing trials from clinical trials registries, conference abstracts, and reference lists of associated reviews. An example of search strategy for PubMed database that combines MeSH terms and free words will be adopted. The search strategy was as follows:

#1 Search: (“Stomach Neoplasms”[Mesh]) OR ((((((((((((((((((Neoplasm, Stomach[Title/Abstract]) OR (Stomach Neoplasm[Title/Abstract])) OR (Neoplasms, Stomach[Title/Abstract])) OR (Gastric Neoplasms[Title/Abstract])) OR (Gastric Neoplasm[Title/Abstract])) OR (Neoplasm, Gastric[Title/Abstract])) OR (Neoplasms, Gastric[Title/Abstract])) OR (Cancer of Stomach[Title/Abstract])) OR (Stomach Cancers[Title/Abstract])) OR (Gastric Cancer[Title/Abstract])) OR (Cancer, Gastric[Title/Abstract])) OR (Cancers, Gastric[Title/Abstract])) OR (Gastric Cancers[Title/Abstract])) OR (Stomach Cancer[Title/Abstract])) OR (Cancer, Stomach[Title/Abstract])) OR (Cancers, Stomach[Title/Abstract])) OR (Cancer of the Stomach[Title/Abstract])) OR (Gastric Cancer, Familial Diffuse[Title/Abstract]))
#2 Search: (“xihuang” [Supplementary Concept]) OR ((xi huang[Title/Abstract]) OR (xihuangwan[Title/Abstract]))
#3 Search: (((((((((randomized controlled trial[Title/Abstract]) OR RCT[Title/Abstract]) OR random[Title/Abstract]) OR randomly[Title/Abstract]) OR random allocation[Title/Abstract]) OR allocation[Title/Abstract]) OR randomized control trial[Title/Abstract]) OR controlled clinical trial[Title/Abstract]) OR clinical trial[Title/Abstract]) OR clinical study[Title/Abstract]
#1 and #2 and #3

### 2.3 Study selection and data extraction

#### 2.3.1 Selection of studies

**Figure 1:**
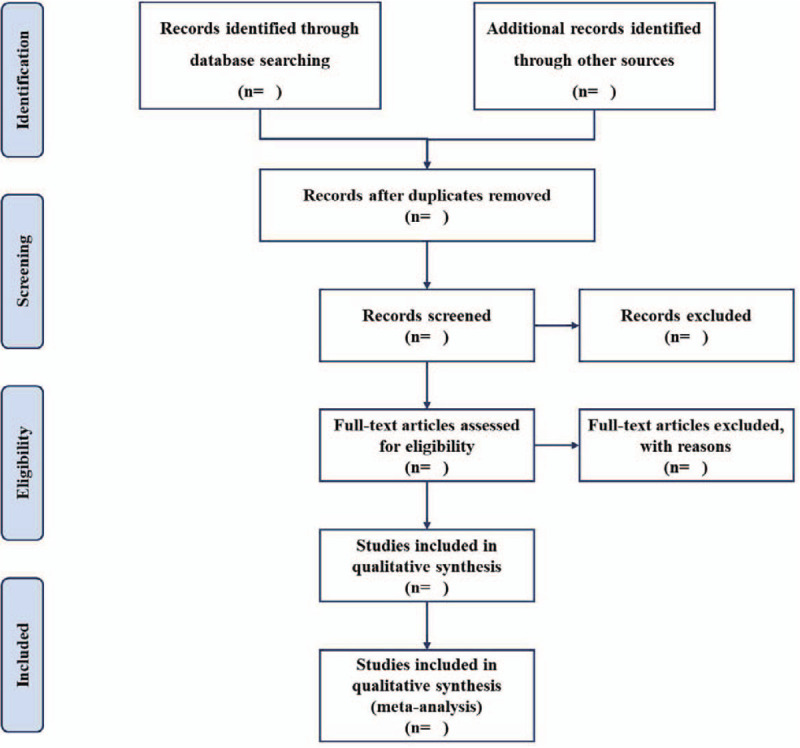
Flow chart of study selection.

After all the documents have been retrieved, the search results were imported into Endnote X9.0 (Thomson Corporation, Connecticut, United States) to eliminate duplicate imported documents. Two trained reviewers independently screened qualified documents by title and abstract. In the next analysis, the 2 reviewers cross-checked the documents, and disagreements in the literature screening, if any, were resolved through discussion. After that, if there are still differences, the third reviewer will make the decision. The flow process of filtration is shown in a PRISMA flowchart in Figure 1. After the screening, if the number of the documents included is more than 3, then a meta-analysis will be conducted, but if it is less than 3, then a descriptive analysis will be conducted.

#### 2.3.2 Data extraction and management

Two researchers will extract relevant data independently with the standardized sheet recommended by the Cochrane Handbook of Systematic Reviews of Interventions. The data of those qualified articles will be export to Microsoft Excel, which includes basic information (registered identification, first author, author unit, country, and publication year), research design (sample size, random sequence generation, allocation concealment, analysis of the data, processing of missing data, blinding of the participants, blinding of the outcome measurement, and blinding of the assessors), participants (disease, age, disease stage, and diagnostic criteria), details of treatment and comparison (e.g., delivery methods, dosage, and frequency), outcomes (outcome measurement), adverse events, conflicts of interest, and other essential information. If unclear or missing data is examined, we will contact primary authors to achieve it whenever possible. If there is any dispute in the data extraction process, it will be submitted to a third researcher for processing. Once the extraction is complete, the 2 researchers will check with each other to ensure the accuracy of the data.

#### 2.3.3 Assessment of risk of bias

We will detect publication biases and poor methodological quality of mall studies using funnel plots if 10 or more studies are included in the meta-analysis. Begg and Egger egression test will be utilized to detect the funnel plot asymmetry.^[14–16]^ If publication bias existed, a trim-and-fill method should be applied to coordinate the estimates from unpublished studies, and the adjusted results were compared with the original pooled risk ratio.^[17]^

#### 2.3.4 Synthesis of data

RevMan 5.2 (Cochrane, London, UK) and stata 14.0 software (Stata Corporation, College Station, TX, USA) will be applied for statistical analyses. For dichotomous variables, the risk ratio (RR) will be applied to analyze. For continuous variables, a mean difference (MD) or a standard mean difference will be used for analysis. MD will be used when the treatment outcome was measured by the same scale. Standard mean difference will be used when the treatment outcome was measured by different scales in different studies. The confidence intervals for both dichotomous and continuous variables will be set to 95%.

#### 2.3.5 Assessment of heterogeneity

To assess the statistical heterogeneity of evidence, the Chi-Squared (*X*^2^) test and the inconsistency index (*I*^2^) statistic.^[18]^ If *P *≥* *.05 and *I*^2^* *≤* *50%, it suggests that no statistical heterogeneity is observed between subgroups, and the Mantel-Haenszel fixed model will be employed for meta-analysis. If *P* < .05 and *I*^2^ > 50%, it is considered that there is great heterogeneity between the studies, and the random effect model will be used. Subgroup analysis, meta- regression, or descriptive analysis will be used for heterogeneity analysis.^[19]^ The results will be showed in tables and figures when the quantitative synthesis is not suitable.

#### 2.3.6 Subgroup analysis

If the necessary data are available, in the case of high heterogeneity, we will conduct subgroup analysis according to the region of the studies, age, stage of the subjects, types of treatments, and different outcomes. We will evaluate the credibility of the subgroup analysis in term of the guidance. If quantitative synthesis is not appropriate due to substantial heterogeneity, then systematic review will be conducted and the results will be displayed in tables and figures.

#### 2.3.7 Sensitivity analysis

A sensitivity analysis for primary outcomes will be performed to evaluate the robustness of the review conclusions if feasible. We will exclude each study included in the analysis. Then we will reanalyze and compile the data. The difference between the reobtained effects and the original effects will be compared.

#### 2.3.8 Assessment of reporting bias

When there are sufficient studies available (normally more than 10 studies), we will check the reporting bias using funnel plot and Egger regression test.^[16,20]^*P* < .05 is considered to have publication bias.

#### 2.3.9 Grading the quality of evidence

The Grading of Recommendations Assessment, Development, and Evaluation will be used to assess the quality of evidence. It contains 5 domains (bias risk, consistency, directness, precision, and publication bias). And the quality of evidence will be rated as high, moderate, low, and very low.

### 2.4 Patient and public involvement

No patient was involved in this protocol for systematic review and meta-analysis.

### 2.5 Ethics and dissemination

The data used in this systematic review will be collected from published studies. Based on this, the study does not require ethical approval. This systematic review will be disseminated through a peer-reviewed publication.

## 3 Discussion

GC, a common cancer with high morbidity and mortality worldwide, requires continuous exploration for new treatment methods and concepts.^[21]^ Xihuang pill is a famous Chinese patent medicine for the treatment of gastric cancer in clinical practice, and a series of clinical studies have been conducted on it. However, there is no systematic review related to Xihuang pill for GC published. In this study, we will conduct systematic review and meta-analysis to provide more evidence on the effectiveness and safety for it. These findings may provide helpful guidance for clinicians in the treatment of gastric cancer.

## Author contributions

**Conceptualization:** Junwei Wang, Xin Tan.

**Data curation:** Junwei Wang, Daorui Hou, Jian Xiong, Yahui Peng.

**Formal analysis:** Yahui Peng, Jian Xiong.

**Funding acquisition**: Xin Tan.

**Investigation:** Jian Xiong, Yahui Peng, Daorui Hou.

**Methodology:** Junwei Wang, Daorui Hou, Xin Tan, Lu Xiong.

**Project administration**: Xin Tan.

**Resources:** Junwei Wang, Jian Xiong, Yahui Peng.

**Software:** Junwei Wang, Daorui Hou, Jian Xiong.

**Supervision**: Xin Tan.

**Writing – original draft:** Junwei Wang, Daorui Hou, Jian Xiong.

**Writing – review & editing:** Junwei Wang, Daorui Hou, Jian Xiong, Xin Tan, Lu Xiong.
